# Supplementary material for: Peptide Toxins from Antarctica: The Nemertean Predator and Scavenger Parborlasia corrugatus (McIntosh, 1876)
Source: Toxins (Basel). 2024 Apr 30;16(5):209. doi: 10.3390/toxins16050209 (PMC11126048; doi:10.3390/toxins16050209)
Supplement: Supplementary file 1 [file toxins-16-00209-s001.zip › toxins-2905511-supplementary.pdf]

Supplementary Materials: Peptide Toxins from Antarctica: The Nemertean Predator and Scavenger *Parborlasia corrugatus* (McIntosh, 1876)

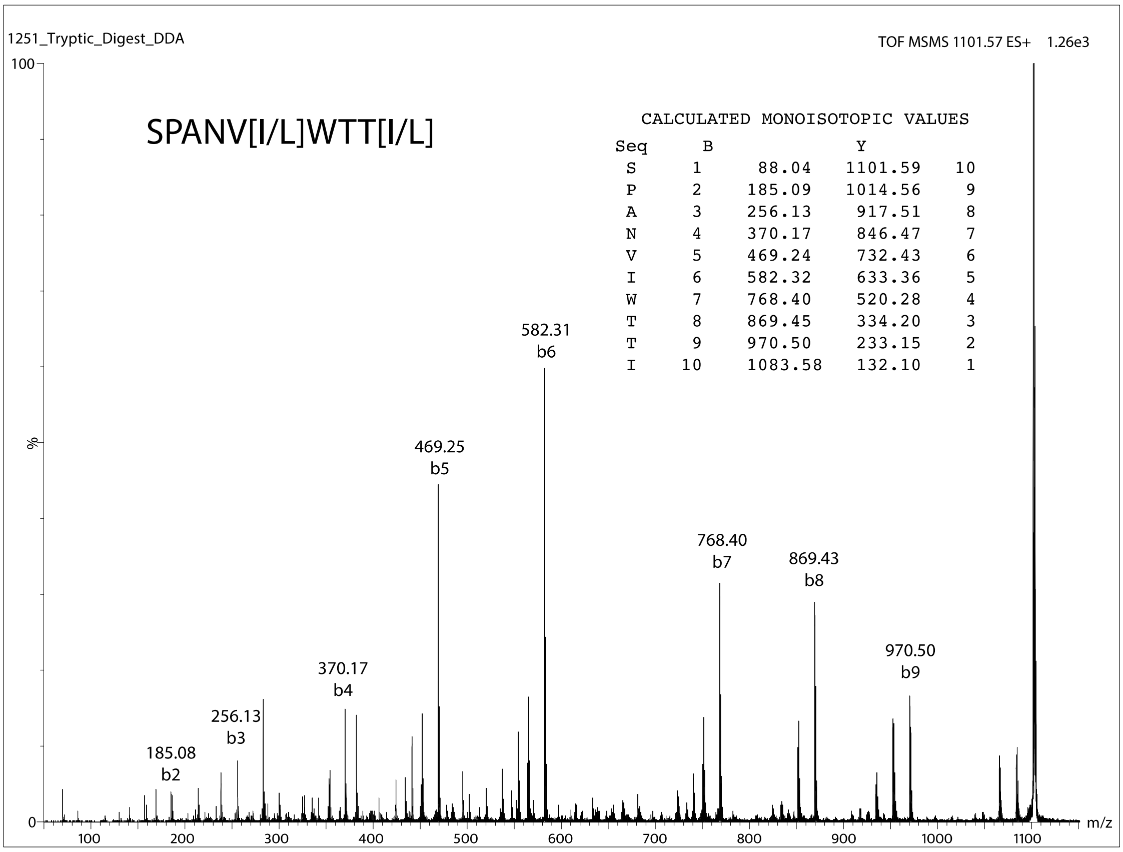

Supplementary Figure S1. De novo sequencing of 1101 fragment. The sequence of the 1101 fragment was found to correspond to SPANV[I/L]WTT[I/L] (positions 75-84) by MSMS de novo sequencing.
